# Development of a Dye-Based Device to Assess Poultry Meat Spoilage. Part I: Building and Testing the Sensitive Array

**DOI:** 10.1021/acs.jafc.0c03768

**Published:** 2020-10-30

**Authors:** Lisa Rita Magnaghi, Giancarla Alberti, Paolo Quadrelli, Raffaela Biesuz

**Affiliations:** † Department of Chemistry, University of Pavia, Via Taramelli 12, 27100 Pavia, Italy; ‡ Unità di Ricerca di Pavia, INSTM, Via G. Giusti 9, 50121 Firenze, Italy

**Keywords:** optical sensors, acid−base dyes, cheap devices, building of sensor array, reproducibility of homemade device

## Abstract

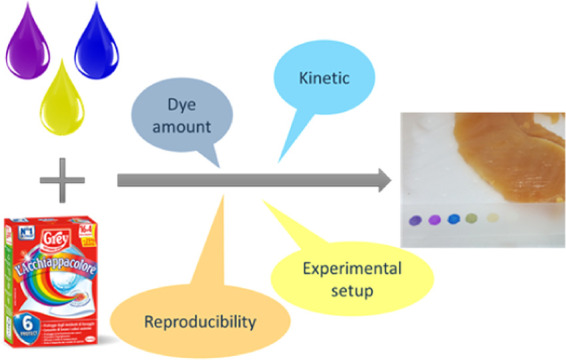

The rationale behind the material and dye selection and the investigation of the properties of a solid-phase sensor array designed for following chicken meat spoilage is presented, having in mind that the final target must be the naked eye identification of the degradation steps. The device is obtained by fixing five acid–base indicators, *m*-cresol purple (1), *o*-cresol red (2), bromothymol blue (3), thymol blue (4), and chlorophenol red (5), and a sensing molecule specific for thiols, 5,5′-dithiobis(2-nitrodibenzoic acid), called Ellman’s reagent, (6) on a commercial cellulose-based support. The dimensions of the sensor and the amount of dye sorbed on the solid are carefully studied. The preparation protocol to get reproducible sensing materials is established, based on the kinetic study and the color change investigation. The material stability and the capacity of changing color, according to the acid–base properties of the dyes, are tested. The sources of uncertainty, coming from the technique employed for signal data acquisition and treatment and from the intrinsic variability of the spots based on the commercial support, are established. The highest variability does not come from photo acquisition by a mobile phone, the effect of the illumination equipment, the partial least-squares (PLS) model employed to assess the amount of dye sorbed into the solid but from the variability of different spots and was found equal to 10%. The uncertainty is adequate for final employment since it is referred to as replicates under different conditions that are definitively judged almost always identical by naked eye evaluation, which is our last target for assessing a change of the colors associated with spoilage.

## Introduction

The shelf life of a meat product is the period during which it keeps its qualitative characteristics. Bacteria accompanying meat spoilage produce unpleasant odors, flavors, discoloration, gas, and slime. Several ignored alterations deserve attention from food business operators and competent authorities.^1^

The World Health Organization (WHO) and the Food and Agriculture Organization (FAO) of the United Nations (UN) assert that we waste at least one-third of the total food produced for human consumption each year. The primary reason for food rejection is due to meat spoilage, and it depends on all changes giving unacceptable products for the consumer.^1^

Nowadays, society is becoming ever more focused on the importance of diet for health. Hence any issue relating to food safety has a significant impact on consumer behavior and official rules. Simultaneously, consumers prefer high quality and easy-to-prepare products, which are safe and minimally processed, with fewer additives and ingredients, and with an extended shelf life. The meat industry is, therefore, looking for, on the one hand, emerging technologies that can achieve these processing and storage goals, and on the other hand, new effective methods to determine meat degradation.^1,2^

Commonly, the methods employed to evaluate meat spoilage require instrumental or microbiological analysis and sensory evaluation. These techniques have some drawbacks in common: they are all destructive and expensive, and they require skilled people and have a long-time response.^2^ Moreover, in the case of sensory evaluation, the practical use of the human nose as an odor assessment tool in the food industry is limited by the subjectivity of human olfaction. Accordingly, there is a considerable need for an instrument that could mimic this human sense. Although many studies describe some successful applications of electronic nose systems, they still showed limitations in sensitivity to recognize analytes at low concentration and selectivity to identify different compounds.^3^

In the last decade, chemical sensing experienced fast growth in the development of both sensing materials and analytical techniques. Focusing on food control, biosensors, electronic-tongues, electronic-noses, and optical-based sensors have been tested by several research groups in the last years.^4,5^

Colorimetry is a prompt analytical technique, it is relatively simple, and the introduction of universal digital imaging has given it new and promising possibilities. Array-based devices use different cross-reactive sensors that interact with analytes through physical adsorption or chemical reactions and generate a response. The most common optical sensor arrays are based on colorimetric or fluorescence changes originating from the interactions between the chromophore or fluorophore with the analytes.

In this scenario, colorimetric sensor arrays based on naked eye analysis could overcome some limitations of traditional array-based sensors, i.e., electronic-noses, such as the generally low selectivity and the need for electrochemical instrumentation and statistical tools for data analysis. In fact, optical array sensing has demonstrated excellent performance in the detection of different analytes, for example, chemical hazards, medical biomarkers, and food additives.^5^

Colorimetric analysis using sensor arrays has numerous advantages. First, it allows in situ naked eye detection simply based on color change and without sophisticated instruments. Second, thanks to digital imaging, colorimetric sensor arrays provide a simple and efficient approach for the rapid detection and identification of several analytes. Moreover, these devices could be easily miniaturized and allow multiple analyses, and last but not least, they show advantages such as good selectivity, excellent sensitivity, nondestructive and nonexpensive analysis, and a fast response.^5^

In the field of meat control, colorimetric sensor arrays, based on chemo-responsive dyes, shows great potential in food “odor visualization”; indeed, these devices can change color after reaction with volatile compounds formed in the headspace of packaged meats.^2,6−9^ The drawback of the current devices for practical application will be discussed in detail in part II.

Based on the literature, the use of several pH indicators having different log *K*_a_ values to cover a wide pH range around neutrality, and a selective probe for thiols ensure a reliable methodology for food degradation evaluation. In fact, for each degradation step, the distinctive volatile by-products have a different acid–base behavior resulting in slight changes in the acidity of the headspace over meat samples during its spoilage. Moreover, from protein catabolism, some volatile sulfur compounds are released by bacteria as the spoilage continues.^10−12^

For this reason, a dye-based colorimetric array obtained by fixing, on a solid support, five different dyes that changed their color in a pH range around neutrality and a reagent for thiols was used as the starting point for the development of our device. Further discussion on this topic could be found in part II. As the solid support, we selected the Color Catcher, a product of the washing powder market, distributed in Italy by Gray, a partner of the Henkel company, and in England by Dylon. We have already used it for the development of other colorimetric sensors since it is an excellent and cheap sorbent for the selected dyes.^13,14^

This first paper focuses on the preparation and characterization of the array. The version presented here, despite looking basic and unsophisticated, require systematic work, with an estimate of uncertainty sources, to prove and assess to have a device ready to work in a real case for the final purpose, sensing of chicken meat spoilage on real samples.

## Materials and Methods

### Methods and Chemicals

All reagents were of analytical grade. *m*-Cresol purple (1), *o*-cresol red (2), bromothymol blue (3), thymol blue (4), chlorophenol red (5), and Ellman’s reagent (6) were purchased from Carlo Erba or Sigma-Aldrich.

A Dylon Color Catcher was bought from a local supermarket.

UV–vis spectra of solutions and solid supports were recorded using a JASCO V-750 spectrophotometer.

Pictures of the array were taken by a Smartphone Samsung Galaxy S7; a portable light-emitting diode (LED) lightbox was used to guarantee the reproducibility of the photos (PULUZ, Photography Light Box, Shenzhen Puluz Technology Limited); a picture of the lightbox is reported in the Supporting Information, Figure 1S.

GIMP software (open-source program, https://www.gimp.org/) was employed to collect the RGB values; this software was preferred to others since it allows defining the area of the photo to be analyzed, usually here the entire spot, and gives the average values of the RGB triplet for each sample.

### The Sensitive Part of the Sensors

**Figure 1:**
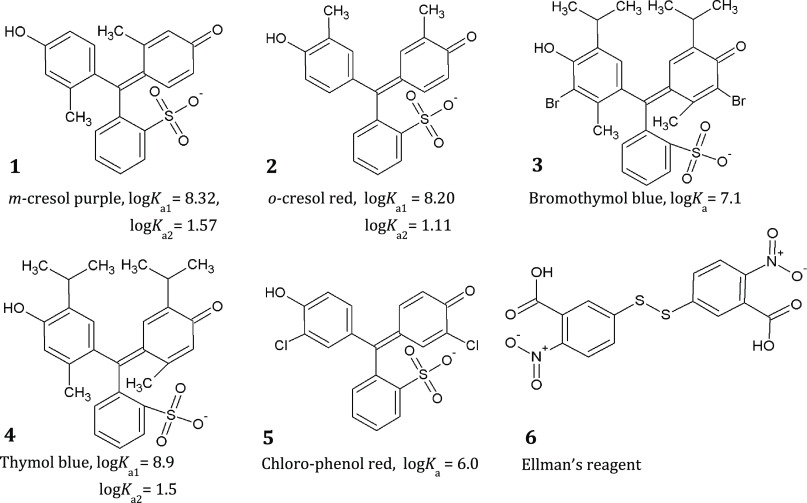
Chemical formula of the six dyes employed as sensing moieties and their log *K*_a_ values.

Figure 1 shows the dyes employed in the array. The first five, *m*-cresol purple (1), *o*-cresol red (2), bromothymol blue (3), thymol blue (4), and chlorophenol red (5), are acid–base indicators and are reported with their log *K*_a_ values, as found in the literature.^15,16^ The sixth is Ellman’s reagent (5,5′-dithiobis(2-nitrodibenzoic acid, DTNB)) (6): a molecule with two electron-deficient phenyl groups linked by a sulfhydryl bridge. Reacting with thiols, DTNB undergoes a transsulfuration reaction which involves the reduction of the sulfhydryl group and the release of a highly chromogenic product, 5-thio-2-nitrobenzoate (TNB), with an intense absorption band at 412 nm.^14^ All of these molecules present a permanent negative charge. For convenience, in the following, numbers from 1 to 6 in Figure 1 are always associated with the same dye.

### The Solid Support of the Array

As solid support, we chose Color Catcher, here named under the acronym CC, a product of the washing market, distributed in Italy by Gray, in England by Dylon, partners of Henkel Company, purchasable in any supermarket.

CC and similar products have been present in the market for several years. They have become successful for their ability to prevent color run during the washing operation. A Color Catcher package contains 16 sheets of the same dimension 11 × 25 cm^2^, and it costs about 3/4€. The sheets appear rather rigid (96% of dry substance), but once wetted, they become soft, very similar to fabric.

The CC exhibited sequestration properties toward molecules and ions when released by clothes, even in the presence of surfactants and fabric softeners. Since tissue dyes are often anionic, we tested it as an anion exchange device.

The chemical–physical characterization of the solid-phase was already discussed in two previous papers, where Alizarine RedS and Ellman’s reagent were employed as the sensitive part of two different sensors for hard metal ions and sulfur compounds in natural waters, respectively.^13,14^

For the present research, different solid supports were also tested such as conventional anionic exchange membranes, which are too expensive for this stage of the research, still explorative, and other products of the washing market, which are characterized by different fabric textures. Once more, the CC was preferred for several reasons. Not only is it incredibly cheap but also the preparation of the sensor has several advantages: it is effortless and environmentally friendly, it involves quick reactions and assures good reproducibility. Even if it is not be the final candidate for the intelligent label, we intend to realize, and it constitutes the best choice as solid support for this stage of the study.

Ellman-CC characterization was already discussed in our previous work,^14^ while for the other receptors, it would be described and discussed below.

### Selection of the Ideal Amount of Dye Sorbed on CC

To select the proper amount, CC bare pieces of 2 × 2.5 cm^2^, were equilibrated overnight with 20 mL of dye solution, at a known concentration, ranging from 0.1% *q*_max_ to 10% *q*_max_ (*q*_max_ = 0.4 mmol g^–1^, is the maximum sorption capacity of CC for the dyes).^13,14^ After this, the sensors were exposed, in a sealed box of 1.5 L, to over 25 mL of 0.001 M NH_3_ solution or 1 M CH_3_COOH, according to their log *K*_a_ values and acid or basic form after sorption. After exposure, the original pH was restored, and these two steps were repeated four times to evaluate both sensitivity and reversibility of the sensors and to select the ideal amount of sorbed dye.

### Sensors Miniaturization and Preparation Procedure

After preliminary results, we decided to miniaturize the sensing units, moving from bare pieces of 2 × 2.5 cm^2^, with a total surface of 5 cm^2^ and an average mass of 0.03 g, to circles of 0.4 cm diameter, with a surface of 0.13 cm^2^ and an average mass of 0.0015 g. They were obtained from the dry CC by punching a hole.

The preparation procedure was updated in terms of volume and concentration of dye solution required: 1 mL of the dye solution, at a proper concentration, was placed in an Eppendorf tube, and the sensing spots were dipped in it and left to equilibrate overnight at ambient temperature, on a stirring plate.

### Partial Least-Squares (PLS) Models for Quantification of Dye Sorbed

The amount of each dye sorbed on the solid spot was quantified directly from the color of the spot. For doing so, a sequence of reference spots with a known amount of dye was prepared.

We selected six concentrations, including the blank samples, the highest concentration being slightly higher than the one selected for building the final sensor. The samples of each concentration level were prepared in triplicates. The solutions were left to equilibrate with the CC spot, as in the previously described procedure. After equilibration, the sensors were placed in the lightbox and photographed. The RGB triplets for each spot were acquired. They constitute the training set for the development of the PLS model. CAT, Chemometric Agile Tool, free software developed by the Gruppo Italiano di Chemiometria della Società Chimica Italiana was employed for this purpose (http://www.gruppochemiometria.it/index.php/software/19-download-the-r-based-chemometric-software). As an example, Table 1 reports the concentration levels of the sequence employed in the case of bromothymol blue.

**Table 1:** Concentration Panel for Building up the Training Set for the PLS Model of Bromothymol Blue

dye concentration (M)
0
9 × 10^–7^
4 × 10^–6^
6 × 10^–6^
9 × 10^–6^
1.8 × 10^–5^

### Kinetic Profiles

Sorption kinetics was investigated using a discontinuous procedure: in 10 independent Eppendorf tubes, CC spots of 0.4 cm diameter were put in contact with 1 mL of dye solution at the concentration generally used for sensor preparation. The Eppendorf tubes were left stirring on a shaking plate at room temperature, and, at a specific time, the spots were separated from the solution and photographed in the lightbox. RGB values were submitted to the PLS model to quantify the amount of sorbed ligand at each time, and kinetic profiles were obtained by plotting the sorbed fraction, *f*, against time.

### Reproducibility Evaluation

In our research, the term “reproducibility” refers to two different aspects of the experimental analysis. At first, the reproducibility of the image acquisition method, which involved the employment of a lightbox and a standard smartphone camera, was evaluated. For this purpose, we took 10 photos of the same array during the day, and the RBG of each sensor was acquired and analyzed.

Then, the reproducibility of the final sensors, which includes, in this case, the variability of the starting material and of the preparation procedure, was investigated. For this purpose, for each pH indicator, 10 independent sensing spots, obtained from different sheets of CC, were prepared as established and analyzed by photo acquisition. The reproducibility was assessed based on the RGB values collected and compared.

The RGB triplets were also submitted to the PLS model. Consequently, for each sensor, the amount of dye sorbed is obtained for all replicates.

## Results and Discussion

### Preliminary Results on CC 2 × 2.5 cm^2^ Sensors

The maximum uptake capacity of the CC as an anion exchanger was determined in our previous research^13,14^ and found to be *q*_max_ = 0.4 mmol g^–1^.

For the present application, even being far from saturation, the determination of the ideal amount of dye to put on CC is crucial. This aspect represents a key point for the performances of the colorimetric sensors. Two conflicting processes must be balanced: on the one hand, the color of the sensing unit must be intense enough so that the color change can be observed by the naked eye. On the other hand, the lower the amount of sensing molecules on the CC, the lower the amount of acid or basic analytes that will produce a complete and homogeneous color change. Consequently, the sensitivity will increase by reducing the amount of dye that concurrently affects the intensity of the color of the sensor. The kinetics of the change from the acidic to the basic form also depends on the amount of dye, being faster with a low quantity of sorbed dye.

The guiding principle in the development of this device was its final application. So we tested the sensors with vapors from dilute solutions of volatile acids or bases, in particular 0.001 M NH_3_ and 1 M CH_3_COOH, to mimic the slight changes in the acidity of the headspace over meat samples during its spoilage. As examples, figures in Section 2S of the Supporting Information show these findings.

**Figure 2:**
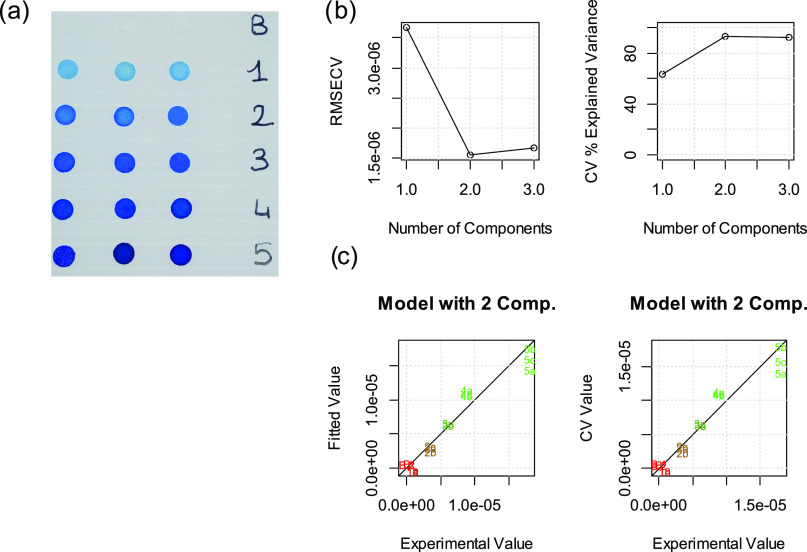
Example of the PLS model for bromothymol blue (3): sensors used as the training set (a); plot of root mean square error of validation (RMSEV) and explained variance in cross-validation as a function of the number of latent variables (b); experimental values vs the predicted values according to the proposed model, on the left, and the same vs CV on the right (c).

The sensitivity and reversibility were tested by decreasing the amount of ligand sorbed on the CC. In Figure 2S.1, the results after the exposition of the CC charged with different amounts of dyes to 0.001 M NH_3_ are shown. Figure 2S.1 shows some attempts with 1 M acetic acid solution vapor response.

These systems were all reversible, and the colors changed from the acidic to the basic form and vice versa as long as we tried. Conversely, we observed that the sensors with the highest amount of sorbed dye (around 5% *q*_max_) required the longest reaction times and did not present a uniform coloration, even after a long equilibration time. On the other hand, the sensors with the lowest amount of dye (*q* = 0.1% *q*_max_) showed rapid reactions. However, the coloration was too faded to be eligible for naked eye analysis. The optimal concentrations were finally selected and are reported in Table 2.

**Table 2:** Experimental Conditions for the Bare CC pieces 2 × 2.5 cm^2^ for Sensor Preparation (uptake solution volume 20 mL)

		[dye] (M)	dye sorbed (mmol)	*q* (mmol g^–1^)	% *q*_max_
1	*m-*cresol purple	7 × 10^–6^	1.40 × 10^–4^	0.005	1.2
2	*o*-cresol red	4 × 10^–6^	8.00 × 10^–5^	0.003	0.7
3	bromothymol blue	9 × 10^–6^	1.80 × 10^–4^	0.006	1.5
4	thymol blue	8 × 10^–6^	1.60 × 10^–4^	0.005	1.3
5	chlorophenol red	7 × 10^–6^	1.40 × 10^–4^	0.005	1.2

However, rapidly we moved to test real samples, and we selected poultry meat as a tester. A common selling tray containing 300 g was used and different sensors were suspended over the meat for 48 h. Some of the sensors reacted with volatile spoilage by-products during the analysis. Unfortunately, even if a color change took place, it was never uniform but presented different hues.

This experimental evidence could be explained assuming that the volatile by-products released during spoilage were not concentrated enough to provoke the complete reaction of the entire amount of sensing molecules sorbed on the CC. We could not overcome this problem by further decreasing the dye amount sorbed since the final color would be too faded to allow naked eye analysis.

Consequently, the sensors prepared with the described procedure were still not suitable for real sample analyses. Therefore, we moved from bare pieces of 2 × 2.5 cm^2^ to circles of 0.4 cm diameter, as described in the Materials and Methods section, and we continued with this unit device for the following. The final amount of sorbed dye was recalculated according to the new dimension of the sensing units, and the final values are reported in Table 3, standardizing the preparation procedure in an Eppendorf tube.

**Table 3:** Experimental Conditions for the CC Spot Sensor Preparation

		dye concentration (M)	dye sorbed (mmol)
1	*m*-cresol purple	7 × 10^–6^	7 × 10^–6^
2	*o*-cresol red	4 × 10^–6^	4 × 10^–6^
3	bromothymol blue	9 × 10^–6^	9 × 10^–6^
4	thymol blue	8 × 10^–6^	8 × 10^–6^
5	chlorophenol red	7 × 10^–6^	7 × 10^–6^
6	Ellman’s reagent	2.4 × 10^–5^	2.4 × 10^–5^

### Characterization of Miniaturized CC Sensing Spots

The last steps of the development included the investigation on the sorption kinetics and the reproducibility of the final material. For both these analyses, we need to quantify the dye present in the solid. It was not possible to follow the kinetics from the decreasing coloration of the dye solution since the dyes were too dilute for UV–vis spectroscopy analysis, as done elsewhere.^13^ The sensitive spots were also too small to be inserted into the sample-holder for recording the UV–vis spectra of the CC directly, as already successfully done in other cases.^13−18^ It is possible to quantify, for each pH indicator, the amount of dye sorbed in the solid-phase directly from the color of the spot, developing a dedicated PLS model. The RGB triplets for the known amounts of dye constitute the training set, the color of spots collected during the kinetic experiments, or in any other case, constitutes the test set to predict the unknown concentration. All of these experiments were carried out under the same circumstances, so further testing the procedure for the PLS model is redundant here.

For the PLS model, three independent sensors for each concentration level were analyzed, and an example of the standardization is shown in Figure 2, where the panel of sensors prepared in the case of bromothymol blue (3) is also shown, together with PLS outputs. The PLS models for the other sensors are reported in the Supporting Information, see Section 3S.

In this case, two components were considered significant and used to develop the model, and the model was then used to assess the amount of sorbed dyes in the following experiments.

**Figure 3:**
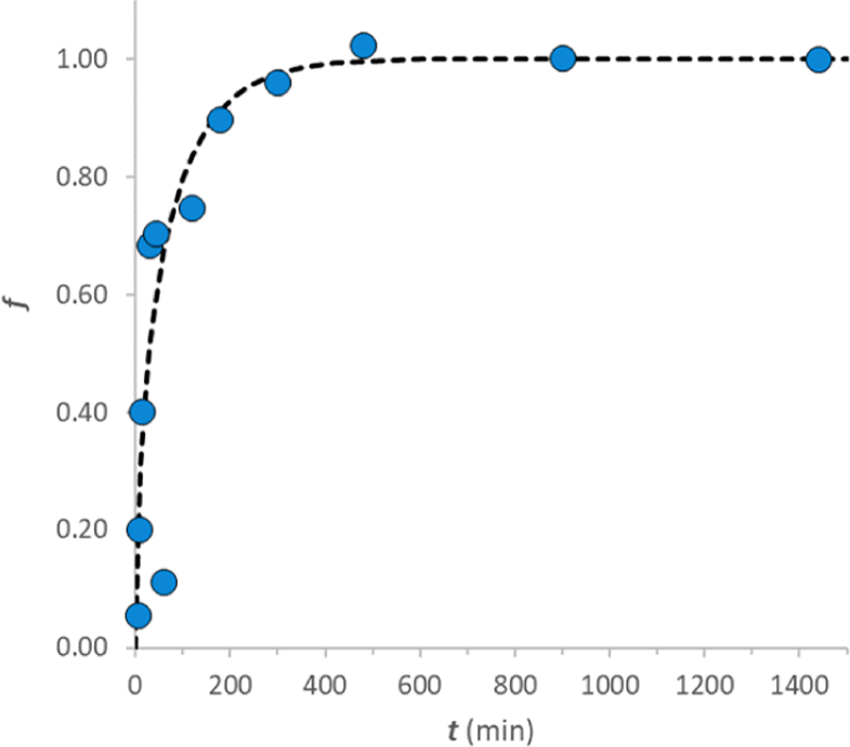
Sorption kinetic profile of bromothymol blue (3) on CC. 10 spots of 0.4 cm diameter (0.0015 g) CC kept in contact with 1 mL of 9 × 10^–6^ M dye solution. Fitting by the HPDM model:^12^ solid line film diffusion (FD limiting stage) and dashed line particle diffusion (PD limiting stage).

Kinetic experiments were performed to establish the suitable timing for obtaining stable and reproducible sensors. Figure 3 reports the kinetic sorption profile of bromothymol blue (3) on CC. The homogeneous particle diffusion model (HPDM model)^19^ was applied for data fitting. These data were not enough to determine which kinetic process limits the sorption of the dye, but further investigations on this point are out of the scope of our study. Our goal from the kinetic profile is to assess the equilibration time. Since the complete sorption process took around 6–7 h and similar conclusions were drawn with other dyes, the procedure employed for obtaining the sensitive material, which provides for overnight equilibration, is definitively adequate.

In any case, the kinetic profiles for other dyes, obtained as described, with the fitting based on the HPDM model are reported in the Supporting Information, Section 4S.

### Reproducibility Tests of Mobile Phone Photos in the Lightbox

**Figure 4:**
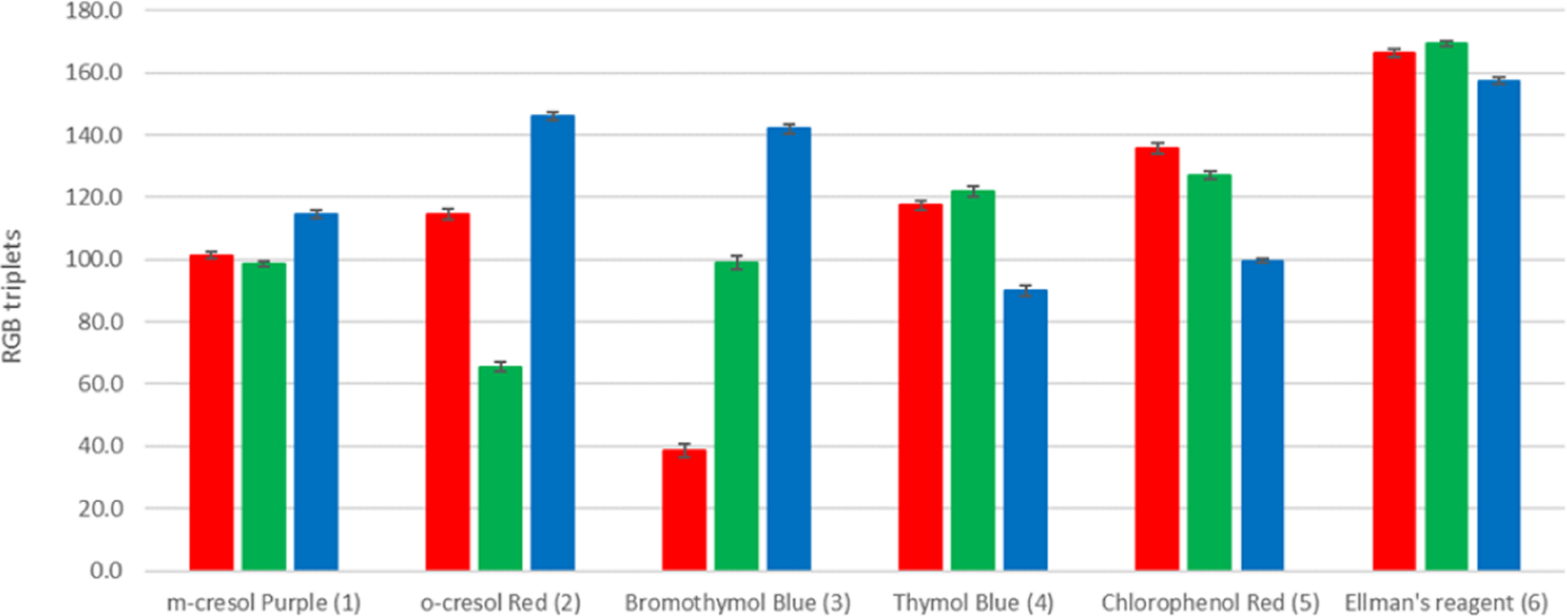
RGB triplets on 10 pictures collected for the same array during the 1 day lab time.

The results testing the reproducibility of the photo acquisition setup, including the lightbox, mobile phone camera, and experimental setup are shown in Figure 4. We considered the array as-prepared for the final application, consequently made of the six dyes blocked on CC. The average values of RGB triplets with standard deviation in the bars are shown in Figure 4.

The overall relative standard deviation is 1.5%. It is worth noting that the contribution to the overall variability due to illumination and photo acquisition is definitively low. As a consequence, for the purpose of this research, the reproducibility of this acquisition method was judged to be satisfactory (Table 4).

**Table 4:** Average Value and Standard Deviation of R, G, B, and the Predicted Concentration

		*R*	*G*	*B*	predicted concentration (M)
1	*m*-cresol purple	64(5)	43(4)	113(7)	1.03(2) × 10^–5^
2	*o*-cresol red	115(4)	20(3)	181(4)	3.6(6) × 10^–6^
3	bromothymol blue	6(3)	52(7)	140(9)	1.3(1) × 10^–6^
4	thymol blue	72(14)	76(9)	52(6)	9.9(7) × 10^–6^
5	chlorophenol red	115(5)	38(4)	196(4)	6.6(9) × 10^–6^

The flash mode was evaluated to acquire the same photos, but it was discarded because of higher variability (around 4%) and more attention required in positioning the camera. Again, it must be highlighted that the photos, when observed by the naked eye, are identical.

### Reproducibility Tests of Different Replicates of Each Sensor

**Figure 5:**
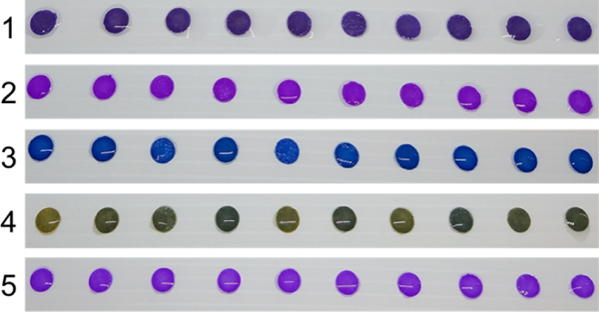
Ten different sensor units per reactive dye, as-prepared for the reproducibility test (concentrations of dye solution are reported in Table 2, and always referred to the volume of 1 mL).

The reproducibility that considers the variability of the different sensors and of the preparation was then investigated, analyzing 10 independently prepared sensors per dye. In Figure 5, the photographs of these sensors are displayed.

**Figure 6:**
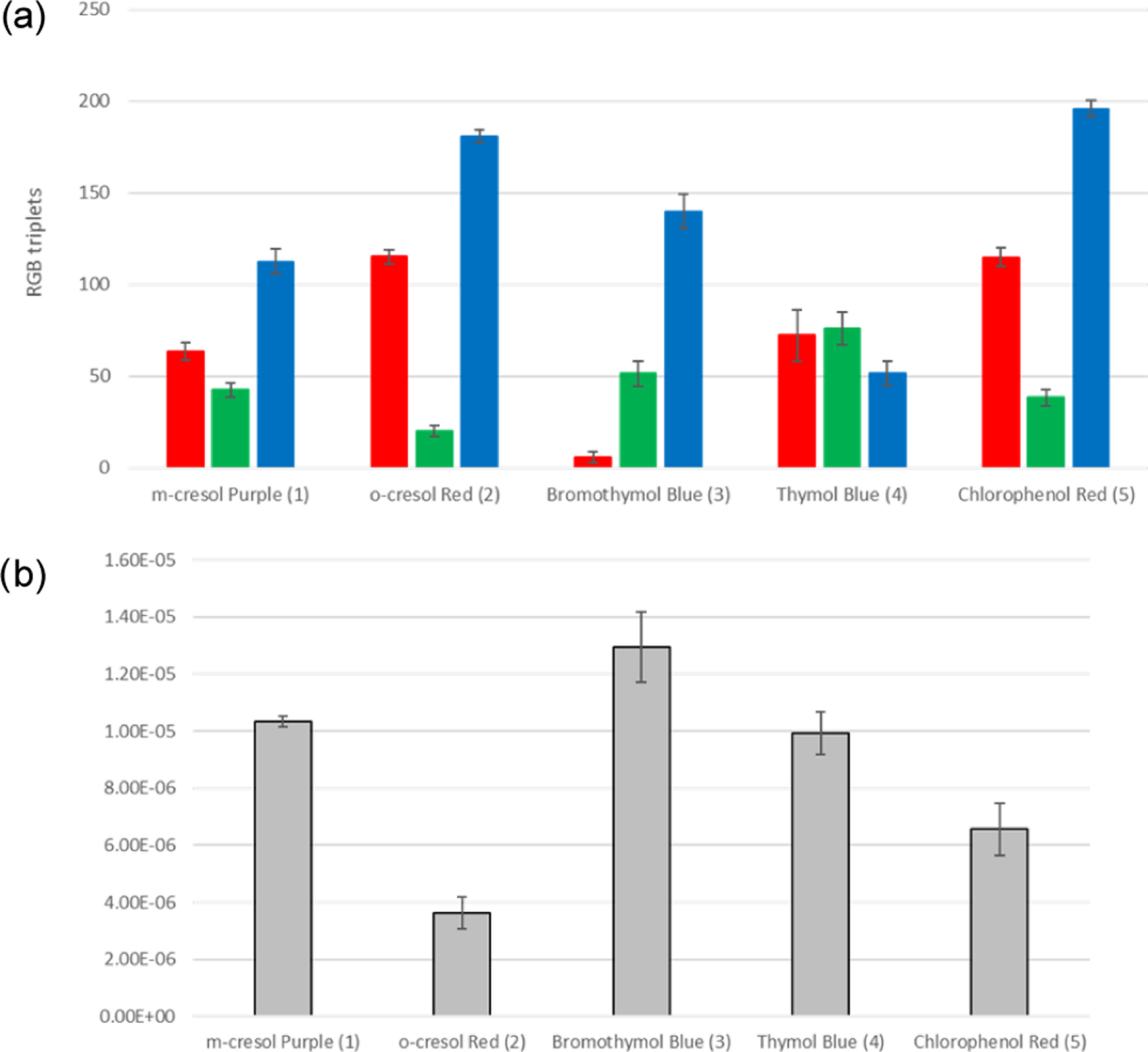
Histograms representing the average value of RGB triplets (a) and predicted concentration (b), referred to the reproducibility tests. In both graphs, the error bars represent the standard deviation values, reported in Table 4.

The average RGB values of 10 replicates are reported in Figure 6a, with standard deviation. The overall relative standard deviation is around 10%, as expected, higher than that on the replicates of the photos, demonstrating that no significant variance is introduced by the acquisition mode and confirming its suitability.

We can also project the RBG triplets into each of the PLS models built for kinetic studies, determining the concentration of sorbed dye in each replicate (referred, for simplicity, to the concentration in the original 1 mL of solution). The predicted concentrations are displayed in Figure 6b. The reproducibility is again around 10%. Once more, no further variability is produced by the quantification method. In this case, we can also compare the average concentrations with the nominal ones, and we find no significant differences at a confidence level of 95% for *o*-cresol red and chlorophenol red, at a confidence level of 90% for others.

In any case, observing Figure 5, the color on the 10 replicates hardly ever look different if analyzed by the naked eye. This result is encouraging for the development of an in-field application. The variability of the system is definitively acceptable, having in mind that in the final application, the color difference to be assessed by the naked eye will be between the protonated and deprotonated color of the sensors, consequently definitively glaring.

### Final Chameleon Array Ready for a Real Case Study

The final version of the array suitable to successfully react in in-field application was finally produced as described above, see Table 3. The CC spots with the different dyes were dried and kept in a sealed container ready for use. It was verified that the colors of dyes-CC do not change for months when kept in a closed container.

**Figure 7:**
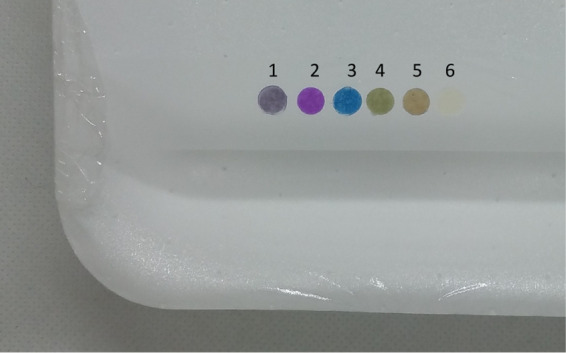
Array prototype ready for the real sample analysis.

When needed, they were placed on a stripe of Scotch 3M Magic Tape in the usual order and put over the tray to expose the free side to the inner part of the packing. The final setup on an empty tray is shown in Figure 7.

It must be underlined that, except for Scotch 3M Magic Tape, other adhesives like glue or standard tape were not suitable since they all release acid substances that change the color of the dye into the acidic one.

Under this setup, the array does not change any of its colors if exposed in a closed system over pure water and water with phosphate buffer 0.008 M, indicating that the change of colors, as we will observe during spoilage monitoring, is caused by substances present in the headspace only if the meat is present.

Notice that the images used for analyses reported in Figure 4 were obtained with the array in this final arrangement, placed on the Magic Tape covered with low-permeability polyethylene plastic film.

The array, at this stage, was always prepared with the six indicators. It is prepared as described, fixing the dye sensors on the stripe, in the order from one to six, always kept from left to right.

### Perspectives

In this first part of the work, despite looking basic and unsophisticated, we demonstrated that every aspect of the array construction was analyzed in detail, from the assessment of the dye sorption kinetics to the source of uncertainty of the different aspects of its construction. It may seem redundant but by describing it step by step, we assure that the results obtained applying it in the case study will be definitively reliable. The second part of the work is focused on sensing chicken meat spoilage under home conditions, placing the device in the common selling tray as described above, and monitoring the degradation of meat samples from the purchasing to the complete spoilage at different temperatures. The sensor responses were used to follow naked eye analysis of the degradation, to model this process by principal component analysis (PCA), and to perform a very first attempt of classification by linear discriminant analysis (LDA); the obtained models were eventually validated by instrumental measurements both on meat samples and on headspace composition.

The final product is definitively cheap, simple, based on smooth and, clean chemistry. All of these features, in our opinion, represent the dye-CC array strong point for possible implementation in the food market and, for this reason, a patent based on this idea has been submitted and more recently the extension to WIPO PCT.^20,21^

## Supporting Information Available

White box (1S); selection of dyes, acid/basic form, and concentration of dyes over the CC (2S); PLS models (3S); and Kinetic profiles (4S) (PDF)

## Abbreviations

WHO: World Health Organization
FAO: Food and Agriculture Organization
UN: United Nations
DTNB: Ellman’s reagent (5,5′-dithiobis(2-nitrodibenzoic acid)
TNB: 5-thio-2-nitrobenzoate
CC: Color Catcher
PLS: partial least square regression
HPDM: homogeneous particle diffusion model
PCA: principal component analysis
LDA: linear discriminant analysis
