# Supplementary material for: Development of a Dye-Based Device to Assess Poultry Meat Spoilage. Part I: Building and Testing the Sensitive Array
Source: J Agric Food Chem. 2020 Oct 30;68(45):12702–9. doi: 10.1021/acs.jafc.0c03768 (PMC8015209; doi:10.1021/acs.jafc.0c03768)
Supplement: Supplementary file 1 — jf0c03768_si_001.pdf [file jf0c03768_si_001.pdf]

Supplementary section

1S. The white box.

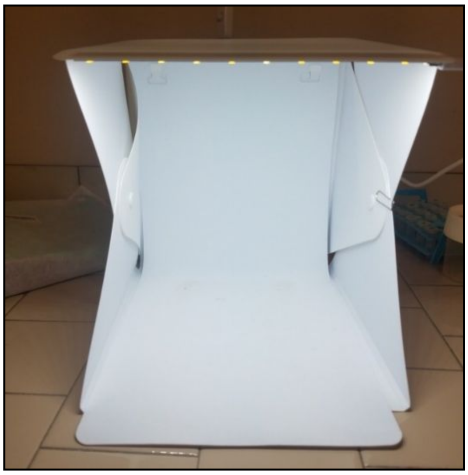

Figure 1S- Picture of the white box employed to take array photographs.

2S. Selection of dyes, acid/basic form, and concentration of dyes over the CC.

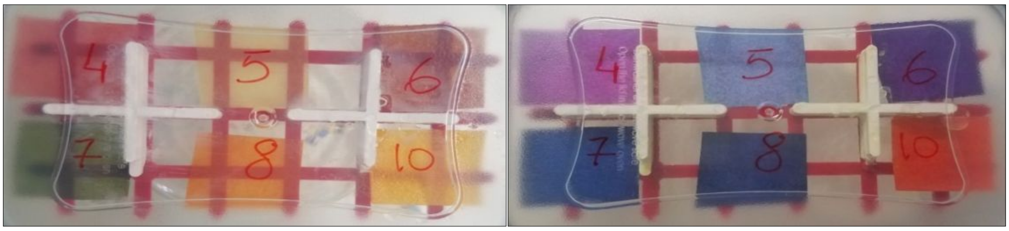

Figure 2S.1: Functionalised CC before (on the left), and after (on the right) exposition to ethylenediamine vapours of 1M solution: 4) Phenol red; 5) *m*-cresol Purple; 6) *o*-cresol Red; 7) Bromothymol Blue; 8) Thymol Blue; 10) Titan Yellow. Ethylenediamine volume 25 mL; sealed box total volume 0.24 L.

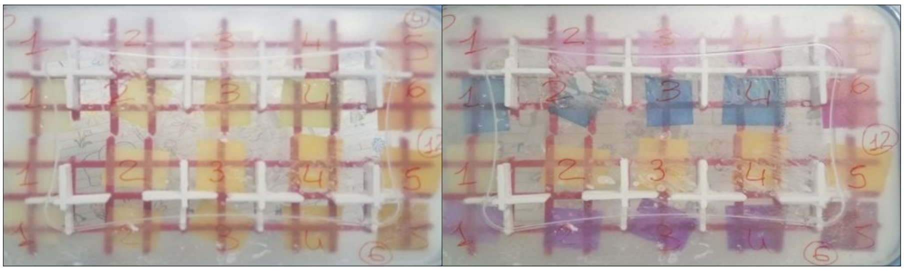

Figure 2S.2: Functionalised CC with increasing dyes concentrations (from around $1 \cdot 10^{-6}$ M to $1 \cdot 10^{-5}$M in V=20 mL), before, on the left, and after, on the right, exposition to vapours of a 0.001M of NH_3 solution: 4) Phenol red; 6) *o*-cresol Red; 7) Bromothymol Blue; 12) Alizarine Yellow R.

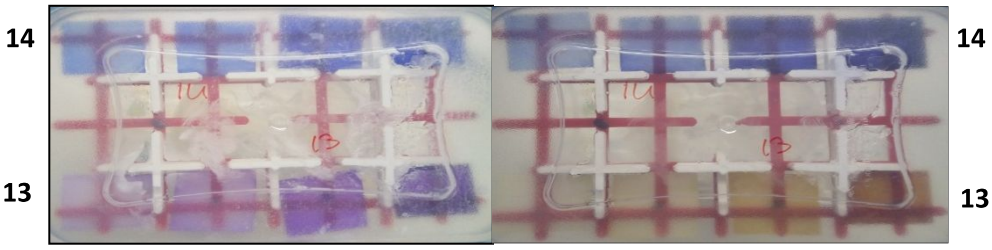

Figure 2S.3: Functionalised CC with increasing dyes concentrations(from around 1 10^{-6} M to 1 10^{-5}M in V=20 mL), before, on the left, and after, on the right, exposition to vapours of a 1M CH_3COOH solution: 13) Chlorophenol Red; 14) Bromophenol Blue.

3S. PLS models

3S.1 m-cresol Purple

Table 1S – Training set for PLS model of *m*-cresol Purple and the related collage of spots imagines.

sample	[Dye](M)
**B**	0
**1**	$8 \cdot 10^{-7}$
	$3 \cdot 10^{-6}$
**3**	$6 \cdot 10^{-6}$
**4**	$8 \cdot 10^{-6}$
**5**	$1.2 \cdot 10^{-5}$

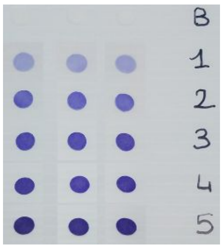

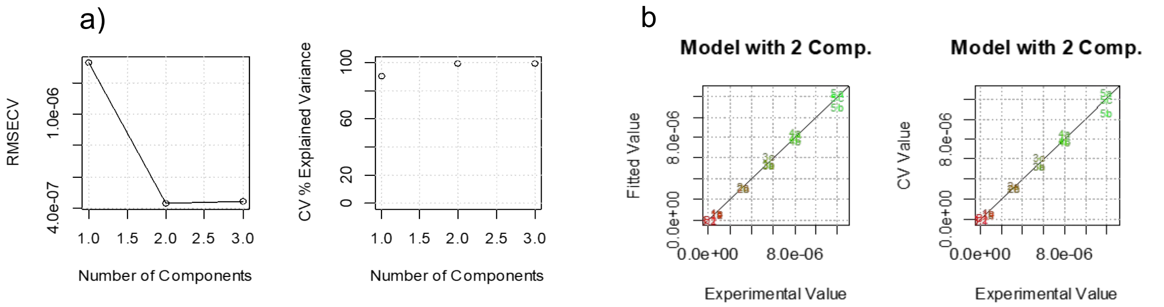

Figure 3S.1 – PLS model for *m*-cresol Purple (1): plot of RMSEV (Root Mean Square Error of Validation) and explained variance in cross-validation as a function of the number of latent variables (a); Experimental values vs the predicted according to the proposed model, on the left, and the same in CV on the right (b).

3S.2 o-cresol Red

Table 2S – Training set for PLS model of *o*-cresol Red and the related collage of spots imagines.

sample	[Dye](M)
**B**	0
**1**	$4 \cdot 10^{-7}$
**2**	$2 \cdot 10^{-6}$
**3**	$3 \cdot 10^{-6}$
**4**	$4 \cdot 10^{-6}$
**5**	$1.0 \cdot 10^{-5}$

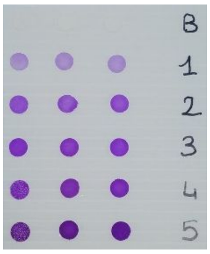

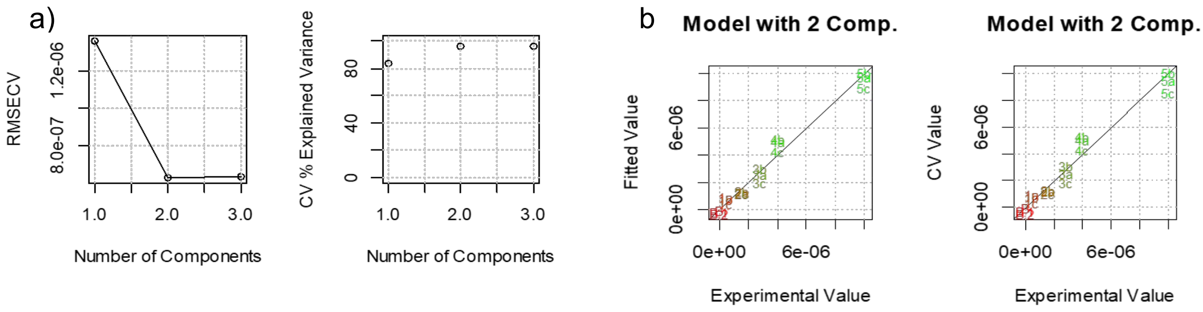

Figure 3S.2 – PLS model for *o*-cresol Red (2): plot of RMSEV (Root Mean Square Error of Validation) and explained variance in cross-validation as a function of the number of latent variables (a); Experimental values vs the predicted according to the proposed model, on the left, and the same in CV on the right (b).

3S.3 *Thymol blue*

Table 3S – Training set for PLS model of Thymol Blue and the related collage of spots imagines.

sample	[Dye](M)
**B**	0
**1**	$8 \cdot 10^{-7}$
**2**	$3 \cdot 10^{-6}$
**3**	$6 \cdot 10^{-6}$
**4**	$8 \cdot 10^{-6}$
**5**	$1.6 \cdot 10^{-5}$

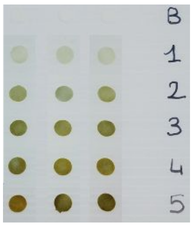

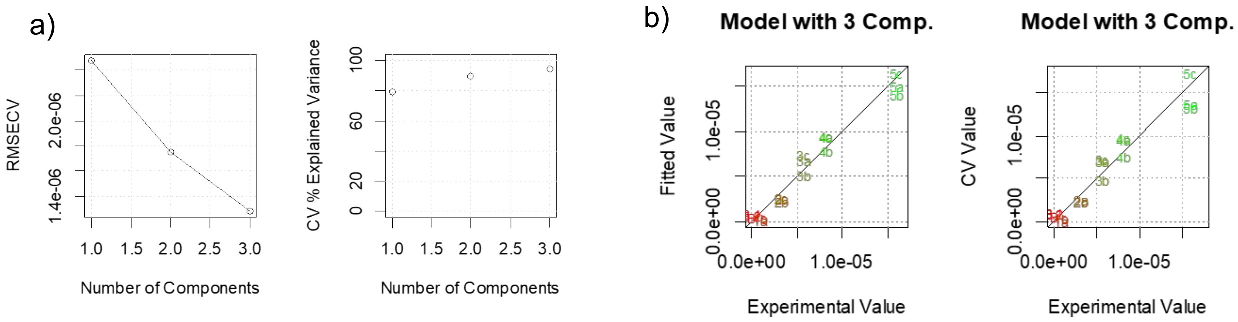

Figure 3S.3 – PLS model for Thymol Blue (4): plot of RMSEV (Root Mean Square Error of Validation) and explained variance in cross-validation as a function of the number of latent variables (a); Experimental values vs the predicted according to the proposed model, on the left, and the same in CV on the right (b).

3S.4 *Chlorophenol red*

Table 4S – Training set for PLS model of Chlorophenol Red and the related collage of spots imagines.

sample	[Dye](M)
**B**	0
**1**	$7 \cdot 10^{-7}$
**2**	$3 \cdot 10^{-6}$
**3**	$5 \cdot 10^{-6}$
**4**	$7 \cdot 10^{-6}$
**5**	$1.4 \cdot 10^{-5}$

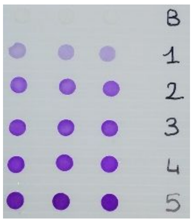

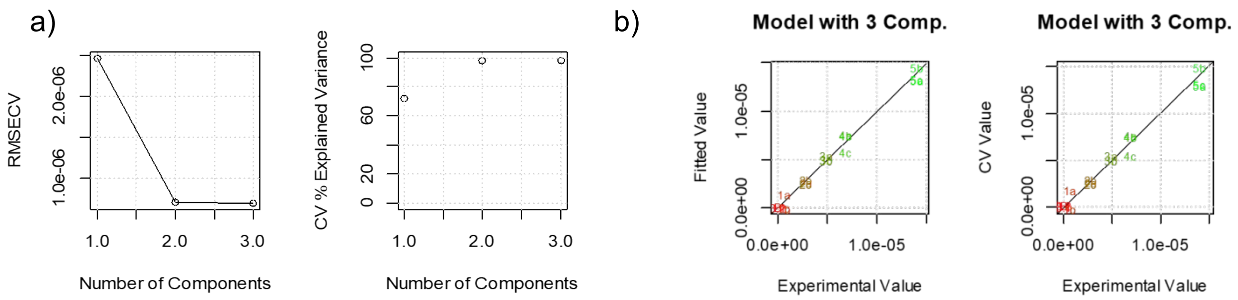

Figure 3S.4 – PLS model for Chlorophenol Red (5): plot of RMSEV (Root Mean Square Error of Validation) and explained variance in cross-validation as a function of the number of latent variables (a); Experimental values vs the predicted according to the proposed model, on the left, and the same in CV on the right (b).

4S. Kinetic profiles

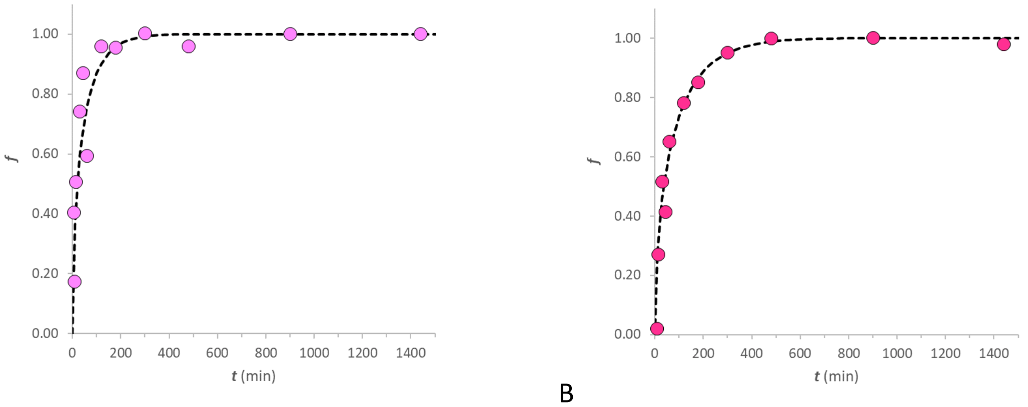

A B

Figure 4S.1- Kinetic profiles of *m*-cresol Purple (1) **(A)** (*V*=1 mL, C_i=6.69x10^{-6} M) and *o*-cresol Red (2) **(B)** (*V*=1 mL, C_i=4.02x10^{-6} M)

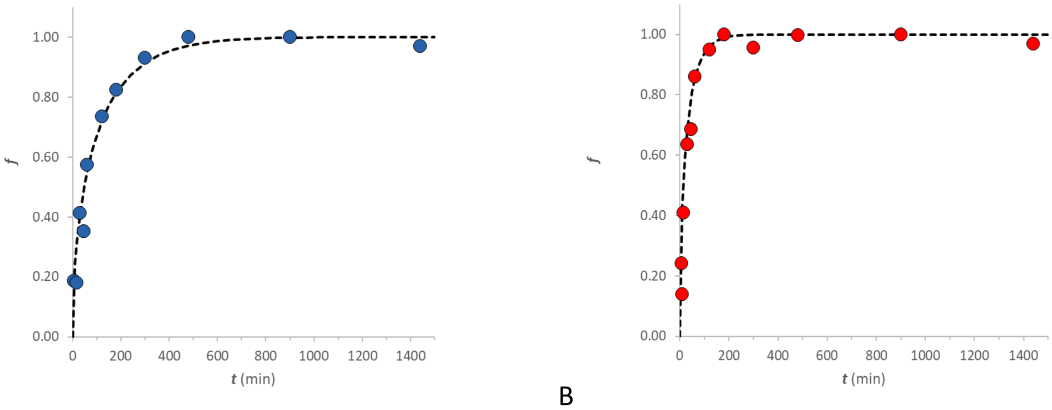

A B

Figure 4S.2.- Kinetic profiles of Thymol blue (4) **(A)** (*V*=1 mL, C_i =8.12x10^{-6} M) and Chlorophenol red (5) (B) (*V*=1 mL, C_i =6.95x10^{-6} M)
